# Physical Performance After Acute Myocardial Infarction: Gait, Frailty, and Cardiovascular Prognosis

**DOI:** 10.1007/s11883-026-01480-6

**Published:** 2026-11-09

**Authors:** Cássia da Luz Goulart, Leandro Franzoni, Luca Correale, Leonardo A. Peyré-Tartaruga

**Affiliations:** 1 Human Locomotion Laboratory (LOCOLAB), Department of Public Health, Experimental and Forensic Medicine, University of Pavia, Pavia, Italy; 2 Hospital de Clínicas de Porto Alegre, Universidade Federal do Rio Grande do Sul, Porto Alegre, Brazil; 3 Unidad Académica de Biofísica, Facultad de Medicina, Universidad de la República, Montevideo, Uruguay

**Keywords:** Acute Myocardial Infarction, Gait Speed, Physical Performance, Frailty, Cardiac Rehabilitation

## Abstract

**Purpose of Review:**

Physical performance and frailty have emerged as potential markers of physiological reserve and cardiovascular vulnerability after acute myocardial infarction (AMI). This review examines the prognostic significance of gait speed, lower-extremity physical performance, frailty, and sarcopenia after AMI and acute coronary syndrome (ACS) and discusses their underlying pathophysiological mechanisms and clinical implications.

**Recent Findings:**

Slow gait speed, impaired Short Physical Performance Battery (SPPB) scores, and frailty are associated with mortality, rehospitalization, heart failure, and adverse cardiovascular events after AMI or ACS. Gait speed and SPPB may provide incremental prognostic information beyond conventional cardiovascular risk assessment and established risk scores. These associations may reflect an interconnected cardiac–vascular–muscle axis involving reduced cardiac reserve, vascular dysfunction, impaired skeletal muscle perfusion, sarcopenia, and physical deconditioning. Importantly, emerging evidence suggests that targeted cardiac rehabilitation may improve gait speed, muscle strength, and overall physical performance in vulnerable patients.

**Summary:**

Physical performance represents a clinically relevant and potentially modifiable dimension of post-AMI cardiovascular vulnerability. Incorporating gait speed, SPPB, and frailty assessment into conventional risk stratification may improve patient phenotyping and identify individuals requiring more intensive rehabilitation and follow-up. Further prospective studies are needed to standardize assessment strategies and determine whether improving physical performance translates into better cardiovascular outcomes.

**Graphical Abstract:**

**Figure:**
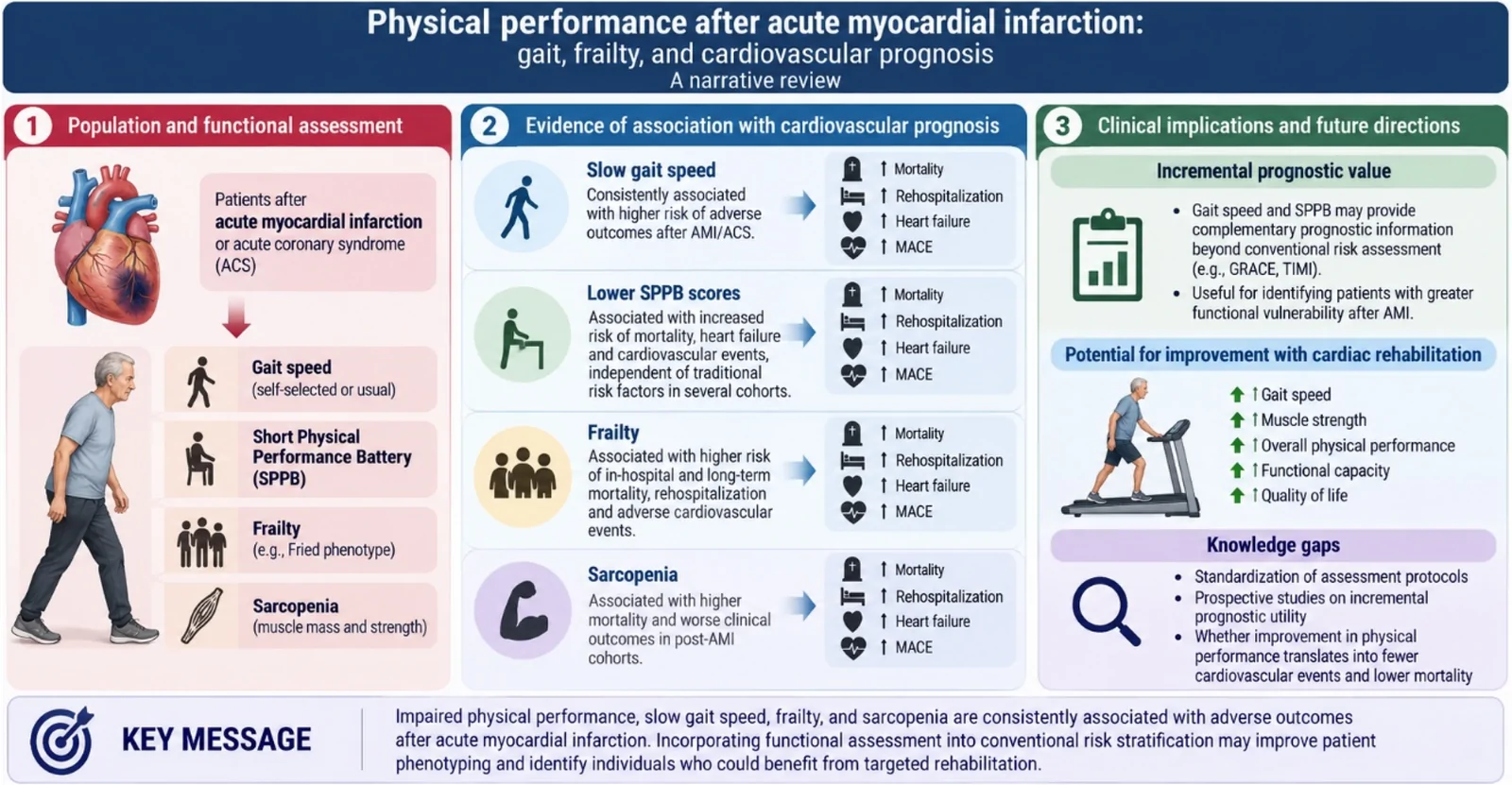
Physical performance, frailty, and cardiovascular prognosis after acute myocardial infarction. Schematic summary of the evidence linking impaired physical performance, including reduced gait speed, lower Short Physical Performance Battery (SPPB) scores, frailty, and sarcopenia, with adverse clinical outcomes following acute myocardial infarction (AMI) or acute coronary syndrome (ACS). These measures may provide complementary prognostic information beyond conventional cardiovascular risk assessment. Exercise-based cardiac rehabilitation may improve functional performance; however, whether these improvements translate into reduced cardiovascular events or mortality remains uncertain. Further prospective studies are needed to establish standardized assessment strategies and determine the clinical utility of integrating functional measures into post-AMI risk stratification and rehabilitation planning.

## Introduction

Acute myocardial infarction (AMI) remains one of the major clinical manifestations of atherosclerotic cardiovascular disease (ASCVD) and an important cause of death, recurrent cardiovascular events, hospitalization, and disability. Contemporary reperfusion strategies, pharmacological secondary prevention, and comprehensive cardiac rehabilitation have markedly improved survival after acute coronary syndrome (ACS) [1–3]. As survival increases and the population presenting with AMI becomes progressively older, cardiovascular care increasingly needs to address not only recurrent ischemic events but also functional recovery, independence, frailty, and quality of life [4–9].

Risk stratification after AMI traditionally incorporates age, hemodynamic status, electrocardiographic findings, biomarkers, renal function, left ventricular function, comorbidities, and scores such as global registry of acute coronary events (GRACE) [10] and thrombolysis in myocardial infarction (TIMI) [11]. These measures quantify important dimensions of cardiovascular risk but do not completely characterize biological age or physiological reserve. ESC guidelines and an American Heart Association scientific statement therefore emphasize the relevance of frailty, multimorbidity, cognitive impairment, and physical function in older patients with ACS [1, 2].

Frailty is characterized by reduced physiological reserve and impaired capacity to maintain homeostasis after a stressor [12, 13]. AMI represents a major physiological stressor capable of revealing or exacerbating previously subclinical vulnerability [4, 5, 14–16]. The classical Fried phenotype includes unintentional weight loss, exhaustion, weakness, reduced physical activity, and slow walking speed, illustrating the close conceptual relationship between frailty and physical performance. Nevertheless, frailty, disability, comorbidity, and chronological age are related but distinct constructs [3, 9, 15].

Physical performance may offer an especially useful perspective because locomotion requires coordinated cardiovascular, vascular, neuromuscular, and metabolic function [4, 6]. Walking depends on adequate cardiac output, peripheral perfusion, skeletal muscle strength and oxidative capacity, balance, cognition, and neurological control. Consequently, impaired gait may function as an integrated phenotype of diminished cardiovascular-functional reserve rather than simply as a manifestation of musculoskeletal disease [17, 18].

This review examines the clinical and prognostic significance of physical performance following AMI, focusing on gait speed, short physical performance battery (SPPB), frailty, and the cardiac–vascular–muscle mechanisms that may underlie functional impairment. We additionally discuss whether these measurements improve cardiovascular risk stratification and whether physical performance represents a potentially modifiable therapeutic target after AMI.

## Methods

A focused narrative review was conducted using PubMed/MEDLINE and complementary searches of clinical guidelines and relevant reference lists. Search terms included combinations of “acute myocardial infarction,” “acute coronary syndrome,” “gait speed,” “walking speed,” “physical performance,” “Short Physical Performance Battery,” “frailty,” “sarcopenia,” “muscle strength,” “cardiac rehabilitation,” “mortality,” “rehospitalization,” and “cardiovascular prognosis.” Priority was given to studies directly evaluating AMI or ACS populations, including prospective and retrospective cohorts, randomized controlled trials, systematic reviews, meta-analyses, and major cardiovascular society guidelines. The literature search covered studies published from database inception to July 2026.

A narrative review approach was selected because the available literature is clinically and methodologically heterogeneous, encompassing different populations, physical-performance and frailty assessment tools, measurement time points, and clinical outcomes. This approach allowed the integration of prognostic, mechanistic, and rehabilitation evidence relevant to physical performance after AMI. Because literature specifically addressing objective physical performance after AMI remains relatively limited, studies involving broader ACS populations were included when AMI constituted a substantial component of the population or when the study directly evaluated gait, SPPB, or physical frailty. Studies of broader cardiovascular populations were considered selectively for mechanistic interpretation or validation of physical-performance instruments. Evidence was narratively organized according to physical performance and gait, frailty, pathophysiological mechanisms, prognostic implications, and potential reversibility through rehabilitation.

### Clinical Assessment and Diagnosis of Frailty and Gait Impairment

#### Assessment and Identification of Frailty

Frailty is a multidimensional clinical syndrome characterized by reduced physiological reserve and increased vulnerability to stressors. Its identification is particularly relevant among older adults hospitalized for AMI, in whom pre-existing functional limitations, multimorbidity, and acute cardiovascular illness may influence recovery and clinical outcomes. The 2023 European Society of Cardiology (ESC) guidelines for ACS recommend incorporating frailty assessment into the comprehensive evaluation of older patients, although no single assessment instrument has been established as optimal for this population [19, 20].

Several validated instruments are available for identifying frailty. The Fried frailty phenotype comprises five criteria: unintentional weight loss, self-reported exhaustion, low physical activity, slow walking speed, and reduced grip strength. Individuals meeting three or more criteria are classified as frail, those meeting one or two criteria as prefrail, and those meeting none as robust [20]. This approach captures the physical manifestations of frailty but requires direct functional measurements and information regarding habitual activity and recent weight changes.

The Clinical Frailty Scale (CFS) provides an alternative approach based on clinical judgment of overall fitness, functional independence, and dependence in daily activities. It uses a nine-point ordinal scale ranging from very fit to terminally ill, with scores of five or higher generally indicating frailty. The FRAIL scale is another brief screening instrument comprising fatigue, resistance, ambulation, illnesses, and weight loss. Scores range from zero to five, with scores of one or two indicating prefrailty and scores of three or more indicating frailty [19].

In patients hospitalized for AMI, the timing of frailty assessment requires consideration. Acute symptoms, hemodynamic instability, bed rest, and hospitalization-related deconditioning may temporarily impair physical performance and lead to overestimation of pre-existing frailty. Therefore, assessments should distinguish baseline functional status from acute illness-related limitations whenever possible. When performance-based testing is not feasible, clinical scales informed by the patient’s usual pre-admission functional status may be more practical. Frailty classification should be interpreted alongside cardiovascular disease severity, comorbidities, cognitive status, and functional independence rather than used as an isolated determinant of clinical management [19, 21].

#### Assessment of Gait Impairment and Lower-extremity Physical Performance

Gait impairment may manifest as reduced walking speed, impaired balance, altered gait pattern, or difficulty initiating and maintaining independent ambulation. In the context of AMI, gait assessment can provide information regarding functional vulnerability, although reduced walking speed alone does not establish the underlying cause of gait dysfunction.

Usual gait speed is among the simplest objective measures of physical performance. It can be assessed by asking the patient to walk at a comfortable, self-selected pace over a standardized distance, commonly four meters, with walking speed calculated by dividing distance by time and expressed in meters per second (m/s). A usual gait speed below 0.8 m/s is frequently used to identify slow walking performance in older adults and has been described as a marker of frailty in ambulatory patients with ACS. Nevertheless, this threshold should not be interpreted as a universally validated diagnostic cutoff for gait disorders or as an AMI-specific prognostic threshold [6, 19].

The SPPB provides a broader evaluation of lower-extremity function by combining standing balance, usual gait speed, and repeated chair stands. Each component is scored from zero to four, yielding a total score ranging from zero to twelve, with lower scores indicating poorer physical performance. A score of eight or less is commonly used to identify impaired physical performance in sarcopenia assessment, although this threshold was not specifically developed to diagnose frailty or gait disorders following AMI [6].

The Timed Up and Go (TUG) test may complement these assessments by evaluating the ability to rise from a chair, walk three meters, turn, return, and sit down. It captures several components of functional mobility, including transfers, walking, turning, and dynamic balance. However, performance may be influenced by musculoskeletal, neurological, vestibular, and cardiovascular conditions, and no single TUG threshold should be regarded as diagnostic of gait disorders specifically in post-AMI populations [20, 21].

When abnormalities are identified, a comprehensive clinical assessment should consider gait symmetry, step length, cadence, balance, use of assistive devices, muscle strength, sensory deficits, pain, and relevant neurological or musculoskeletal conditions. This distinction is important because slow gait speed may reflect reduced physiological reserve without identifying a specific neurological or orthopedic gait disorder. Consequently, quantitative performance testing should be complemented by clinical examination when the objective is to establish the cause of abnormal gait.

#### Clinical Considerations Following Acute Myocardial Infarction

Frailty and gait assessments should be performed when patients are clinically stable and able to participate safely. During acute hospitalization, the selection of instruments should account for symptoms, mobility restrictions, cardiovascular stability, and the feasibility of standardized testing. Reassessment after discharge or during cardiac rehabilitation may help distinguish persistent functional impairment from transient limitations associated with the acute event [19].

Importantly, frailty, sarcopenia, and gait impairment represent overlapping but distinct constructs. Slow gait speed may contribute to the identification of frailty and the evaluation of sarcopenia severity, but it does not independently confirm either condition. Likewise, an abnormal SPPB score indicates reduced physical performance rather than a specific etiological diagnosis [6, 20].

Overall, validated frailty instruments and standardized functional tests provide complementary information regarding physiological reserve, mobility, and physical vulnerability after AMI. Their use may support a more comprehensive clinical assessment, particularly among older adults. However, the optimal instrument, assessment timing, and prognostic thresholds for post-AMI populations remain incompletely established, and these measures should complement rather than replace conventional cardiovascular evaluation [6, 20].

#### Frailty, Sarcopenia, and Outcomes After Acute Myocardial Infarction

Frailty and sarcopenia are closely related but distinct clinical conditions that may contribute to reduced physiological reserve and adverse outcomes following AMI. Frailty represents a multidimensional state of increased vulnerability to physiological stressors, whereas sarcopenia is a skeletal muscle disorder characterized primarily by reduced muscle strength, with low muscle quantity or quality supporting diagnostic confirmation [5]. Although these conditions frequently coexist, they should not be considered interchangeable, as frailty encompasses broader physiological and functional domains beyond skeletal muscle impairment.

The relationship between frailty and sarcopenia is particularly relevant following AMI, when acute myocardial injury, reduced cardiovascular reserve, physical inactivity, and hospitalization-related deconditioning may aggravate pre-existing limitations in muscle function. Reduced muscle strength and impaired lower-extremity performance may contribute to slower gait speed, difficulties with transfers, reduced exercise tolerance, and loss of functional independence [5]. However, distinguishing pre-existing sarcopenia from muscle deterioration occurring during hospitalization remains challenging, particularly when baseline muscle function and body composition are unavailable.

Dimitriadou et al. [15] demonstrated that frailty was associated with increased in-hospital mortality, prolonged hospitalization, and adverse clinical outcomes. These findings support the clinical relevance of frailty assessment but do not establish the independent contribution of sarcopenia to the observed associations [15].

Hu et al. [22] conducted a systematic review and meta-analysis evaluating the prognostic impact of sarcopenia following myocardial infarction. Four post-MI cohorts involving 939 patients were included in the primary analysis. Sarcopenia was associated with significantly higher all-cause mortality (pooled odds ratio [OR] 2.84; 95% CI 1.85–4.36). In contrast, evidence regarding recurrent myocardial infarction was limited, preventing definitive conclusions regarding the relationship between sarcopenia and recurrent ischemic events [23].

These findings suggest that sarcopenia is an important marker of mortality risk following myocardial infarction. Nevertheless, the limited number of AMI-specific cohorts, differences in diagnostic approaches, and potential residual confounding restrict the interpretation of the available evidence. It remains uncertain whether sarcopenia provides prognostic information independently of frailty, cardiovascular disease severity, nutritional status, and comorbidity burden [23].

Objective physical performance assessments may help characterize the functional manifestations shared by frailty and sarcopenia. Gait speed and the SPPB capture aspects of lower-extremity function, while handgrip strength provides information regarding muscle strength. However, impaired gait speed or a low SPPB score alone does not establish a diagnosis of sarcopenia. Consistent with the revised European consensus on sarcopenia, reduced muscle strength indicates probable sarcopenia, whereas confirmation requires evidence of reduced muscle quantity or quality, and poor physical performance indicates severe sarcopenia [5].

From a clinical perspective, combining frailty screening with muscle strength and physical performance assessments may offer a more comprehensive characterization of functional vulnerability following AMI. Such an approach could help identify patients who require further evaluation of muscle health, nutritional status, and rehabilitation needs. Nevertheless, the clinical benefit of routinely combining these assessments has not been established in prospective post-AMI studies [5–21, 23].

Overall, the available evidence indicates that both frailty and sarcopenia are associated with adverse outcomes following AMI or ACS, with particularly consistent findings regarding all-cause mortality [5–21, 23]. Further prospective investigations are required to clarify their independent and combined prognostic contributions and to determine whether interventions targeting muscle dysfunction and frailty can improve functional recovery and long-term cardiovascular outcomes.

#### Physical Performance After Acute Myocardial Infarction

Physical performance describes the objectively measured capacity to execute functional tasks and differs conceptually from both physical activity and cardiorespiratory fitness. Physical activity reflects behavior, whereas cardiorespiratory fitness reflects the integrated ability to transport and utilize oxygen during exercise. Physical performance instruments evaluate practical mobility-related tasks and can therefore capture physiological vulnerability that may remain undetected by resting cardiovascular measurements or self-reported activity [14, 17, 18].

AMI may impair physical function through multiple pathways. Many patients have reduced reserve before hospitalization because of older age, multimorbidity, diabetes, peripheral arterial disease, obesity, sarcopenia, and physical inactivity. Acute ischemic may further accelerate decline through myocardial injury, inflammation, bed rest, reduced mobility, invasive procedures, anxiety about physical exertion, and hospital-associated deconditioning. These factors are particularly relevant in older patients, in whom even brief periods of immobility can contribute to deterioration in strength and mobility [12–14].

Several instruments can quantify this impairment. Gait speed is appealing because it is inexpensive, objective, requires minimal equipment, and can be completed rapidly. The SPPB evaluates three complementary domains, standing balance, usual walking speed, and repeated chair rises, generating a score from 0 to 12, with lower values representing worse lower-extremity performance. Handgrip strength, timed up and go, sit-to-stand tests, and the 6-min walk test provide additional information regarding strength, mobility, and exercise capacity [14–20].

The multidimensional structure of the SPPB may be particularly valuable after ACS. In a prospective cohort of 402 patients aged ≥ 70 years with ACS, Campo et al. evaluated seven frailty and physical-performance scales before discharge. SPPB provided the greatest incremental prognostic value for both major adverse cardiac and cerebrovascular events and all-cause mortality [22]. Addition of SPPB to GRACE or TIMI scores improved prognostic discrimination, supporting the concept that objective physical function captures information complementary to conventional cardiovascular risk markers [10, 11].

#### Gait Speed as an Integrative Marker of Cardiovascular Vulnerability

Gait speed is arguably the most extensively investigated single physical-performance parameter in cardiovascular disease. Walking requires integrated activity of cardiac, vascular, pulmonary, skeletal muscle, neurological, and cognitive systems. Slow gait therefore represents a nonspecific but potentially powerful indicator of reduced multisystem reserve. In general, older populations, lower gait speed is consistently associated with mortality, cardiovascular disease, disability, hospitalization, and loss of independence [3, 22].

Walking speed should not be interpreted solely as a measure of mobility or lower-extremity function. Self-selected walking speed (SSWS) emerges from the interaction between cardiovascular, ventilatory, metabolic, biomechanical, and neuromuscular constraints. In healthy individuals, the freely selected walking speed is generally close to the optimal walking speed (OWS), at which the metabolic cost of transport is minimized. This convergence suggests that healthy individuals spontaneously select a walking velocity that favors locomotor economy. Accordingly, walking speed has been proposed as a “functional vital sign,” integrating multiple physiological systems into a simple measure of functional status [24, 25].

Cardiovascular disease may disrupt this physiological optimization of walking. Figueiredo et al [26]. provided mechanistic evidence in patients with chronic HF (CHF), a population characterized by impaired cardiovascular reserve and exercise intolerance. Patients with CHF selected a significantly slower walking speed than healthy controls (0.75 ± 0.14 vs. 0.98 ± 0.30 m·s⁻¹; *P* < 0.01). Importantly, whereas healthy controls selected the walking speed associated with the lowest cost of transport, patients with CHF selected a speed that was metabolically less economical than faster walking speeds. However, the ventilatory equivalent for carbon dioxide (V̇E/V̇CO₂) was lowest at the self-selected speed in both groups. These findings suggest that, in cardiovascular disease, walking speed may represent a behavioral adaptation to cardiopulmonary limitation: patients may select a slower speed not to minimize total metabolic energy expenditure, but rather to reduce ventilatory demand [34].

Beyond absolute walking speed, the relationship between the speed selected by the individual and the energetically optimal walking speed may provide additional information about gait functionality. Peyré-Tartaruga and Monteiro [27] proposed the Locomotor Rehabilitation Index (LRI), calculated as 100 × SSWS/OWS, where OWS is estimated from lower-limb length using the principle of dynamic similarity. Conceptually, an LRI approaching 100% indicates convergence between the spontaneously selected and theoretically optimal walking speeds, whereas lower values indicate increasing divergence between habitual locomotion and energetic optimization. This approach incorporates body size and locomotor energetics into the interpretation of gait and may therefore provide information not captured by absolute gait speed alone.

Matsuzawa et al. prospectively evaluated 472 patients with STEMI and demonstrated a pronounced gradient of cardiovascular risk according to walking speed. Cardiovascular events occurred in 36.7% of patients in the slowest gait-speed tertile compared with 3.2% in the fastest tertile. Each 0.1-m/s increase in gait speed was independently associated with a 29% lower hazard of cardiovascular events, and gait speed improved the C-statistic of a clinical model from 0.703 to 0.786 [8].

The TRIUMPH registry provided complementary evidence during post-discharge recovery. Among 338 adults aged ≥ 65 years assessed one month after AMI, 53.6% had a walking speed < 0.8 m/s. The composite of death or hospital readmission at one year occurred in 35.4% of patients with slow gait compared with 18.5% in those with preserved gait. The association was attenuated after extensive adjustment for clinical comorbidities, suggesting that gait speed partly integrates the cumulative burden of these conditions rather than acting as an isolated risk factor [3, 8].

Long-term observations further support the prognostic significance of gait. Sanchis et al. [36] assessed components of the Fried frailty phenotype in older survivors of ACS followed for a median of 8.7 years. Among the individual Fried components, low physical activity and slow gait independently predicted mortality; impaired gait was associated with a 77% greater adjusted mortality risk. These findings support gait speed as one of the highest-yield components of multidimensional frailty evaluation in patients with coronary disease [36].

Collectively, these studies suggest that gait speed may be interpreted as an integrated functional cardiovascular biomarker. It does not identify a specific pathophysiological abnormality but reflects the cumulative effect of cardiac disease, vascular dysfunction, comorbidity, muscle health, neurological control, and aging. This integration is precisely what may allow gait speed to complement traditional disease-specific prognostic markers [8].

#### Frailty After Acute Myocardial Infarction

Frailty is increasingly recognized as an important determinant of prognosis after ACS [9]. Its prevalence varies substantially according to population, age, clinical setting, and assessment instrument. Earlier systematic reviews documented prevalence estimates ranging widely across ACS cohorts, reflecting the use of the Clinical Frailty Scale, Fried phenotype, Edmonton Frail Scale, FRAIL scale, deficit-accumulation indices, and other instruments [3, 4, 14].

Despite this methodological heterogeneity, the direction of the association with prognosis is remarkably consistent. A meta-analysis of older patients with ACS reported adjusted mortality risks approximately 2.6-fold higher in frail patients and also demonstrated associations with cardiovascular events, major bleeding, and hospital readmission. A subsequent 2025 meta-analysis showed frailty to be associated with an almost threefold greater risk of in-hospital mortality and a greater risk of major bleeding and prolonged hospitalization [37,38].

More recent evidence has strengthened these findings. A 2026 systematic review and meta-analysis comparing frailty tools in ACS found that frailty significantly predicted mortality regardless of the specific instrument used and found no statistically significant evidence that one of the evaluated scales was clearly superior to the others [39]. The authors suggested that practical considerations may therefore appropriately guide instrument selection in acute cardiovascular settings [39].

AMI-specific cohorts produce similar findings. In the FLAGSHIP study, 524 ambulatory patients aged ≥ 70 years hospitalized for AMI underwent physical frailty assessment at discharge. Frailty was independently associated with the composite of all-cause death or heart failure rehospitalization over two years (HR 2.09). Large contemporary datasets using electronic or administrative frailty indices similarly demonstrate graded increases in in-hospital, 12-month, and longer-term mortality following MI as frailty severity increases [40].

Frailty should therefore not be interpreted as synonymous with chronological age. A robust 80-year-old and a frail 80-year-old patient may have markedly different responses to the same ischemic insult. Physical-performance assessment provides an objective means of capturing part of this heterogeneity and may be particularly useful when determining rehabilitation needs, intensity of follow-up, or the broader goals of post-AMI care [40].

#### The Cardiac–vascular–muscle Axis

The relationship between physical impairment and cardiovascular prognosis is probably mediated by multiple interacting biological systems. AMI can reduce cardiac reserve through myocardial necrosis, impaired left ventricular systolic or diastolic function, reduced stroke volume, chronotropic limitation, and autonomic dysregulation. These abnormalities may be clinically subtle at rest but become evident during activities that increase metabolic demand [1, 2].

Vascular dysfunction provides another potential link. ASCVD is systemic, and coronary atherosclerosis frequently coexists with impaired endothelial function, arterial stiffness, peripheral arterial disease, and microvascular dysfunction. Age-related endothelial dysfunction reduces vasodilatory reserve and may compromise exercise hyperemia and skeletal muscle oxygen delivery. The AHA statement on ACS in older adults specifically recognizes age-related endothelial dysfunction, impaired peripheral vasodilation, arterial stiffening, and reduced maximal cardiovascular output as contributors to the reduced physiological reserve of older patients [2].

Skeletal muscle represents a third major component of this pathway. Aging, inflammation, oxidative stress, physical inactivity, reduced perfusion, insulin resistance, nutritional abnormalities, and hospitalization can contribute to loss of muscle strength and mass. Contemporary consensus defines sarcopenia primarily by reduced muscle strength, with low muscle quantity or quality confirming the diagnosis and impaired physical performance indicating greater severity [41].

Sarcopenia is particularly relevant after MI. A 2026 systematic review and meta-analysis including post-MI cohorts found that sarcopenia was associated with an approximately 2.8-fold higher odds of all-cause mortality, with consistent effects during short- and long-term follow-up. Evidence for recurrent MI was substantially weaker and heterogeneous, indicating that sarcopenia currently appears more firmly established as a marker of vulnerability and mortality than as a direct predictor of recurrent coronary thrombosis [42].

Balance may represent an additional component of this phenotype. Postural control integrates muscular strength, neurological processing, vision, vestibular function, blood pressure regulation, and cognition. Orthostatic abnormalities, autonomic dysfunction, polypharmacy, muscle weakness, and deconditioning could plausibly impair balance after AMI. However, compared with gait speed and frailty, balance and falls have been inadequately investigated specifically after AMI, representing an important evidence gap rather than a currently established prognostic marker [43].

**Fig. 1:**
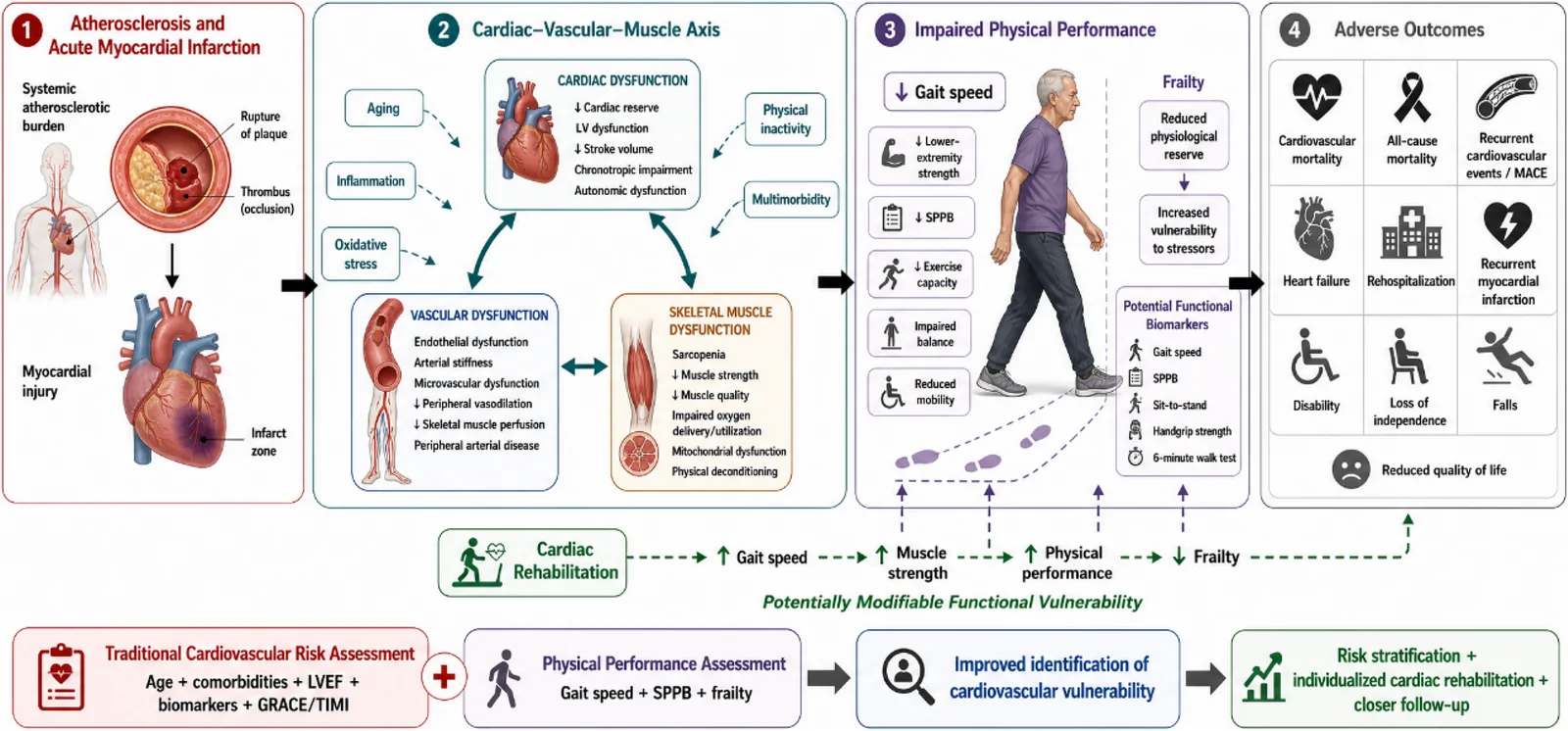
Proposed framework linking acute myocardial infarction, physical performance, and cardiovascular vulnerability. Systemic atherosclerotic burden and myocardial injury may contribute to interconnected cardiac, vascular, and skeletal muscle dysfunction, ultimately resulting in impaired physical performance and frailty. These functional abnormalities are associated with adverse cardiovascular and functional outcomes. Physical performance assessment may complement conventional cardiovascular risk stratification, while cardiac rehabilitation may represent a strategy to modify functional vulnerability. Solid arrows indicate established relationships and dashed arrows indicate proposed mechanisms or emerging evidence. LVEF, left ventricular ejection fraction; SPPB, Short Physical Performance Battery; GRACE, Global Registry of Acute Coronary Events; TIMI, Thrombolysis in Myocardial Infarction; MACE, major adverse cardiovascular events

These observations support a cardiac–vascular–muscle axis linking myocardial injury and systemic atherosclerosis to impaired mobility. Reduced cardiac reserve and vascular dysfunction can compromise peripheral oxygen delivery, while sarcopenia and skeletal muscle metabolic abnormalities reduce the capacity to utilize oxygen and generate force. Their combined clinical expression may include slow gait, reduced chair-rise performance, exercise intolerance, and frailty, which in turn identify patients at increased risk of adverse outcomes (Fig. 1) [7, 16].

#### Physical Performance and Cardiovascular Prognosis

Available evidence suggests that physical-performance measures may provide prognostic information beyond conventional cardiovascular assessment. Matsuzawa et al. [8] demonstrated substantial improvement in event discrimination when gait speed was added to cardiovascular risk factors, BNP, and comorbidity. Campo et al. similarly demonstrated that SPPB improved prediction when added to GRACE and TIMI scores [10, 11].

Collectively, studies evaluating gait speed, physical performance, and frailty after AMI or ACS have consistently demonstrated associations with adverse cardiovascular and functional outcomes, although substantial heterogeneity exists in the assessment tools, timing, and follow-up periods used across studies (Table 1).

**Table 1:** Key studies evaluating gait, physical performance, and frailty after acute myocardial infarction or acute coronary syndrome

Study	Population	*N*	Assessment	Timing	Follow-up	Main finding
Matsuzawa et al., 2013 [8]	STEMI	472	Gait speed	During index hospitalization	2,596 person-years	Each + 0.1 m/s was associated with lower CV-event risk (HR 0.71); C-statistic improved from 0.703 to 0.786
Dodson et al., 2016 [7]	AMI, ≥ 65 y	338	Gait speed, < 0.8 m/s	1 month post-AMI	1 year	Slow gait prevalence 53.6%; death/readmission 35.4% vs. 18.5%
Blanco et al., 2017 [28]	ACS, ≥ 70 y	402	SPPB + multiple frailty scales	Before discharge	1 year	SPPB produced greatest incremental prediction of MACCE and mortality; improved GRACE/TIMI
Alegre et al., 2018 [29]	ACS, ≥ 80 y	532	FRAIL scale	Hospitalization	6 months	Baseline frailty independentlypredicted prognosis
Sanchis et al., 2020 [5]	Elderly ACS survivors	342	Fried phenotype including gait and grip	Before discharge	Median 8.7 y	Gait speed and physical activity independently predicted mortality
Campo et al., 2019/2020 [6, 24]	ACS, ≥ 70 y, SPPB 4–9	235	SPPB, gait, grip strength	1 month post-discharge	6–12 months	Tailored exercise improved SPPB, gait speed and strength
Beigienė et al., 2021 [25]	ACS CR patients, ≥ 65 y	97	Edmonton Frailty Scale + 5-m gait speed	CR admission/discharge	Short-term CR	Rehabilitation improved gait; resistance/balance training improved normalization of slow gait
Ashikawa et al., 2023 [4] – FLAGSHIP	AMI, ≥ 70 y	524	Physical frailty score including functional measures	Discharge	2 years	Frailty predicted death/HF rehospitalization (HR 2.09)
Zong et al., 2023 [30]	AMI, ≥ 75 y	238	Modified Frailty Index	Hospitalization	36 months	Frailty independently predicted MACEs and poorer survival
MIMIC-IV study, 2023 [31]	Critical AMI	5,003	Hospital Frailty Risk Score	Hospitalization	In-hospital/1 year	Increasing frailty predicted both short- and long-term mortality
Chad et al., 2024 [3]	ACS	Systematic review of RCTs	Multiple frailty tools	Various	Various	Very limited randomized evidence targeting frailty; physical performance identified as promising modifiable domain
Heart Lung Circ meta-analysis, 2025 [15]	ACS	14 studies in quantitative synthesis	Validated frailty tools	Hospitalization	In-hospital	Frailty associated with mortality (RR 2.89), major bleeding and longer hospitalization
Cunha et al., 2026 [14]	ACS	Systematic review/meta-analysis	Five frailty scales	Various	Mortality	Frailty predicted mortality; no clear superiority among scales
Hu et al., 2026 [22]	Post-MI	4 MI cohorts, *n* = 939	Sarcopenia	At/shortly after MI	Short/long term	Sarcopenia associated with mortality (pooled OR 2.84)

The table summarizes key observational studies, randomized clinical trials, systematic reviews, and meta-analyses investigating gait speed, objective physical-performance measures, frailty, and sarcopenia in patients with acute myocardial infarction or acute coronary syndrome. Study characteristics, assessment methods, timing of evaluation, follow-up duration, and principal prognostic or interventional findings are presented

*ACS* Acute coronary syndrome, *AMI* Acute myocardial infarction, *CR* Cardiac rehabilitation, *CV* Cardiovascular, *GRACE* Global Registry of Acute Coronary Events, *HF* Heart failure, *HR* Hazard ratio, *MACE* Major adverse cardiovascular events, *MACCE* Major adverse cardiac and cerebrovascular events, *MI* Myocardial infarction, *OR* Odds ratio, *RCT* Randomized controlled trial, *RR* Risk ratio, *SPPB* Short Physical Performance Battery, *STEMI* ST-segment elevation myocardial infarction, *TIMI* Thrombolysis in Myocardial Infarction

Nevertheless, important methodological differences remain. Gait speed has been measured over different distances and at different time points after AMI, while frailty studies employ multiple instruments with different conceptual foundations. Acute illness, vascular access complications, neurological or orthopedic disease, and the ability to ambulate may also influence physical-performance measurements. Therefore, gait speed and SPPB should currently be regarded as complementary prognostic measures rather than replacements for validated cardiovascular risk scores [7, 8].

Unlike many traditional prognostic factors, physical performance is potentially modifiable. The HULK randomized trial enrolled 235 adults aged ≥ 70 years with recent ACS and reduced physical performance. A tailored intervention combining supervised sessions and individualized home exercise resulted in significantly higher SPPB scores at six months, with the benefit maintained at one year. Improvements were particularly evident for walking and chair-rise components [6].

A randomized cardiac rehabilitation study involving older vulnerable and frail ACS patients similarly demonstrated improvement in gait speed during rehabilitation. Addition of resistance and balance training could normalize slow walking speed in a greater proportion of participants than conventional rehabilitation alone, supporting the concept that the locomotor component of frailty can respond to targeted exercise.

### What New Information Is Sufficient to Modify Existing Clinical Practice?

Current evidence supports greater consideration of frailty and objective physical performance in the clinical evaluation of patients recovering from AMI, particularly older adults. Conventional cardiovascular risk assessment primarily focuses on clinical characteristics, hemodynamic parameters, cardiac biomarkers, and ventricular function, whereas frailty and physical performance capture additional dimensions of physiological reserve and functional vulnerability.

Evidence from observational studies indicates that slower gait speed, lower SPPB scores, and frailty are associated with increased mortality, rehospitalization, and adverse cardiovascular outcomes following AMI or ACS. Importantly, studies by Matsuzawa et al. and Campo et al. demonstrated that objective physical performance measures may improve prognostic discrimination when incorporated into conventional cardiovascular risk models. These findings support the clinical relevance of brief functional assessments as complementary components of post-AMI evaluation.

From a practical perspective, gait speed and SPPB are feasible, inexpensive, and reproducible instruments that may help identify patients with reduced functional reserve who would benefit from more comprehensive geriatric assessment, individualized cardiac rehabilitation, and closer evaluation of functional recovery. Furthermore, randomized rehabilitation studies in older patients with ACS have demonstrated improvements in physical performance following structured exercise interventions, supporting the potential reversibility of functional impairment.

Nevertheless, the available evidence does not establish that routine implementation of gait speed or SPPB assessment reduces cardiovascular events or mortality. Similarly, improvements in physical performance during rehabilitation should not be interpreted as direct evidence of reduced cardiovascular risk. Therefore, these instruments should complement, rather than replace, established cardiovascular risk stratification tools or guideline-directed secondary prevention.

### What Does This Review Add to Literature?

This review provides an integrated perspective on the prognostic relevance of gait performance, frailty, and skeletal muscle dysfunction following AMI, highlighting their interconnected contributions to cardiovascular vulnerability. Although previous studies have independently investigated frailty, gait speed, and physical performance in patients with ACS, the present review brings these findings together within a comprehensive cardiovascular–functional framework.

First, this review highlights the potential value of objective physical performance measures, particularly gait speed and the SPPB, as complementary markers of physiological reserve and cardiovascular prognosis. By synthesizing evidence from AMI-specific cohorts and broader ACS populations, it emphasizes that impaired physical performance may provide prognostic information beyond conventional cardiovascular risk factors and established risk prediction models.

Second, the review integrates the relationships among myocardial injury, systemic vascular dysfunction, inflammation, skeletal muscle impairment, and physical deconditioning through a proposed cardiac–vascular–muscle framework. This perspective offers a mechanistic interpretation of why impaired gait and frailty may coexist with increased cardiovascular vulnerability, while recognizing that several proposed pathways remain incompletely established in post-AMI populations.

Third, the review distinguishes frailty from sarcopenia and physical disability, emphasizing their overlapping but nonidentical clinical characteristics. It also incorporates recent evidence on the prognostic significance of sarcopenia after myocardial infarction, broadening the discussion beyond conventional frailty assessment.

Fourth, the review explores the potential relevance of locomotor energetics and the LRI as emerging approaches to characterizing walking impairment. Although these concepts have been investigated in other clinical populations, their application and prognostic validity following AMI remain unexplored. Their inclusion identifies a potential direction for future mechanistic and longitudinal research rather than an established clinical application.

Overall, the principal contribution of this review is the integration of cardiovascular prognosis, objective locomotor assessment, frailty, and skeletal muscle health into a unified framework for understanding functional vulnerability after AMI. This synthesis identifies clinically relevant assessment opportunities, clarifies important limitations of the existing evidence, and establishes priorities for future studies investigating whether functional recovery can contribute to improved long-term cardiovascular outcomes.

### Knowledge Gaps

Despite growing evidence supporting the prognostic relevance of frailty, gait speed, and physical performance following AMI, several important knowledge gaps remain. There is no established consensus regarding the most appropriate instruments, assessment timing, or clinically meaningful thresholds for evaluating physical vulnerability in post-AMI populations. Although gait speed, the SPPB, and multidimensional frailty scales have demonstrated prognostic associations, their comparative performance and incremental clinical utility across different patient populations remain insufficiently characterized. It is unclear whether a single functional assessment or repeated measurements during recovery provide greater prognostic information beyond conventional cardiovascular risk stratification.

The biological mechanisms linking impaired locomotor performance and frailty to adverse cardiovascular outcomes remain incompletely understood. Although myocardial dysfunction, systemic inflammation, endothelial impairment, reduced peripheral perfusion, skeletal muscle abnormalities, and physical deconditioning may contribute to functional vulnerability, their relative contributions and interactions after AMI have not been adequately established. The extent to which these mechanisms reflect pre-existing physiological impairment, acute myocardial injury, or hospitalization-related functional decline also remains uncertain.

The relationships among frailty, sarcopenia, and gait impairment require further clarification. Although these conditions frequently coexist, they represent distinct clinical constructs and may contribute differently to cardiovascular prognosis. Current evidence does not adequately establish whether sarcopenia provides independent prognostic information beyond frailty and conventional cardiovascular risk factors or whether the combined assessment of muscle strength, muscle mass, and physical performance improves the identification of high-risk patients following AMI.

These knowledge gaps highlight the need to distinguish the established prognostic associations of physical impairment from its underlying biological mechanisms, incremental clinical utility, and potential therapeutic implications. Addressing these uncertainties is essential before objective physical performance measures can be incorporated into standardized post-AMI risk assessment pathways or used independently to guide clinical management.

### Future Directions

Future research should move beyond establishing associations between frailty, impaired physical performance, and adverse cardiovascular outcomes toward determining how these assessments can improve clinical decision-making and recovery following AMI. Multicenter prospective studies are needed to establish standardized protocols for evaluating gait speed, the SPPB, and frailty in post-AMI populations. These investigations should determine the optimal timing of assessment, including during hospitalization, at discharge, and throughout post-discharge recovery, while identifying clinically meaningful thresholds and trajectories of functional decline or improvement. Importantly, future studies should evaluate whether these measures provide incremental prognostic information beyond established cardiovascular risk models, including GRACE, left ventricular function, renal function, cardiac biomarkers, and relevant comorbidities. Model discrimination, calibration, external validation, and clinical utility should be systematically examined.

Longitudinal mechanistic investigations should explore the interactions among myocardial injury, systemic inflammation, endothelial dysfunction, peripheral perfusion, skeletal muscle abnormalities, and locomotor performance. Combining cardiovascular imaging, inflammatory biomarkers, vascular function assessments, body composition, muscle strength, and cardiopulmonary exercise testing with objective gait analysis may help characterize the biological pathways underlying post-AMI functional vulnerability. Such approaches could also clarify whether frailty and impaired mobility predominantly reflect pre-existing physiological impairment, acute cardiovascular injury, or potentially reversible consequences of hospitalization and physical inactivity.

Future research should address the implementation of functional assessments in routine cardiovascular care. Pragmatic studies should investigate the feasibility, acceptability, resource requirements, and cost-effectiveness of incorporating brief gait and frailty assessments into post-AMI care pathways. Attention should be given to populations frequently underrepresented in existing studies, including patients with severe frailty, substantial mobility limitations, multimorbidity, and restricted access to rehabilitation services. These investigations should also determine whether assessment-guided rehabilitation and follow-up strategies improve patient outcomes compared with conventional care.

Overall, future investigations should aim to establish a validated and clinically actionable framework integrating cardiovascular risk, frailty, skeletal muscle health, and objective locomotor performance. Such research may clarify whether physical performance should remain primarily a complementary prognostic marker or can also serve as a meaningful target for interventions designed to improve long-term recovery and cardiovascular outcomes following AMI.

### Limitations

Several limitations should be considered when interpreting the evidence synthesized in this narrative review. First, the narrative design does not involve a systematic study selection process, formal risk-of-bias assessment, or quantitative synthesis of effect estimates. Consequently, the findings should be interpreted as a critical synthesis of the available literature rather than as a comprehensive or quantitatively pooled estimate of the prognostic effects of physical performance and frailty following AMI.

Second, substantial clinical and methodological heterogeneity exists across the included studies. Investigated populations differed in age, comorbidity burden, clinical presentation, infarction severity, and treatment strategies. Furthermore, several investigations enrolled patients with ACS rather than exclusively AMI, limiting the direct generalizability of some findings to post-AMI populations. Differences in the instruments used to assess frailty, gait speed, and lower-extremity physical performance, as well as variations in assessment timing, follow-up duration, and outcome definitions, further complicate direct comparisons across studies.

Third, much of the available prognostic evidence derives from observational cohorts, which are inherently susceptible to residual confounding and reverse causation. Slow gait speed reduces physical performance, and frailty may reflect the cumulative effects of aging, multimorbidity, pre-existing disability, cardiovascular disease severity, and hospitalization-related deconditioning. Although several studies adjusted for relevant clinical variables, these factors cannot be completely disentangled. Therefore, the reported associations should not be interpreted as evidence that impaired physical performance independently causes adverse cardiovascular outcomes.

These limitations underscore the need for cautious interpretation of the current evidence. While objective physical performance and frailty assessments appear promising as complementary markers of cardiovascular vulnerability following AMI, their incremental clinical utility, optimal implementation, and potential role in guiding interventions require further prospective validation.

## Conclusions

Current evidence indicates that impaired physical performance, slow gait speed, frailty, and sarcopenia are associated with adverse clinical outcomes following AMI or ACS, particularly mortality, rehospitalization, and heart failure-related events. Gait speed and the SPPB have demonstrated potential incremental prognostic value beyond conventional cardiovascular risk assessment in selected cohorts. These findings support the relevance of objective physical performance measures as complementary indicators of physiological reserve and cardiovascular vulnerability.

The proposed cardiac–vascular–muscle framework provides a physiological rationale for these associations, although the underlying mechanisms and their relative contributions after AMI remain incompletely understood. Evidence from rehabilitation trials in older patients with ACS indicates that targeted exercise interventions can improve physical performance; however, whether these improvements translate into fewer cardiovascular events or improved survival remains uncertain.

Overall, integrating physical performance and frailty assessment with conventional cardiovascular evaluation may contribute to a more comprehensive characterization of patients recovering from AMI. Further prospective studies are needed to establish standardized assessment protocols, determine incremental prognostic utility, and evaluate whether assessment-guided interventions improve long-term cardiovascular outcomes.

## Key References

• Chad T, Koulouroudias M, Layton GR, Fashina O, Sze S, Roman M, et al. Frailty in acute coronary syndromes. A systematic review and narrative synthesis of frailty assessment tools and interventions from randomised controlled trials. Int J Cardiol. 2024;399:131764. https://doi.org/10.1016/j.ijcard.2024.131764. This systematic review highlights the limited randomized evidence addressing frailty in ACS and emphasizes the need for better identification and targeted management of frail patients in contemporary cardiovascular care.
•• Dimitriadou I, Fradelos EC, Skoularigis J, Toska A, Vogiatzis I, Pittas S, et al. Frailty as a prognostic indicator for in-hospital mortality and clinical outcomes in acute coronary syndrome: a systematic review and meta-analysis. Heart Lung Circ. 2025;34:214–24. https://doi.org/10.1016/j.hlc.2024.11.026. This contemporary meta-analysis provides quantitative evidence that frailty is strongly associated with in-hospital mortality and adverse clinical outcomes in patients with ACS, reinforcing frailty as an important dimension of cardiovascular risk assessment.
•• Cunha LN de P, Oliveira SV de, Lazzarin T, Silva LLS da, Minicucci MF, Costa NA. Frailty scale with the best prediction of mortality in individuals diagnosed with acute coronary syndrome: systematic review and meta-analysis. J Geriatr Cardiol. 2026;23:173–83. https://doi.org/10.26599/1671-5411.2026.03.004. This recent meta-analysis demonstrated that frailty is associated with substantially increased mortality in ACS and found no statistically significant difference in mortality prediction among the five frailty scales evaluated, supporting selection of assessment tools according to clinical feasibility and setting.
•• Hu X, Arthur Vithran DT, Parfait MNU, Dou Y, Li H. Prognostic impact of sarcopenia following myocardial infarction: a systematic review and meta-analysis of mortality and recurrent MI. Eur J Prev Cardiol. 2026. https://doi.org/10.1093/eurjpc/zwag329. This meta-analysis provides recent MI-specific evidence linking sarcopenia with all-cause mortality. Across four post-MI cohorts comprising 939 patients, sarcopenia was associated with approximately threefold higher mortality, supporting skeletal muscle dysfunction as an important component of post-MI cardiovascular vulnerability. Evidence for recurrent MI remained limited.

## Data Availability

No datasets were generated or analysed during the current study.

## Abbreviations

ACS: Acute coronary syndrome
AMI: Acute myocardial infarction
ASCVD: Atherosclerotic cardiovascular disease
BNP: B-type natriuretic peptide
CHF: Chronic heart failure
CR: Cardiac rehabilitation
CV: Cardiovascular
ESC: European Society of Cardiology
GRACE: Global Registry of Acute Coronary Events
HF: Heart failure
HR: Hazard ratio
LRI: Locomotor Rehabilitation Index
LVEF: Left ventricular ejection fraction
MACE: Major adverse cardiovascular events
MACCE: Major adverse cardiac and cerebrovascular events
MI: Myocardial infarction
OR: Odds ratio
OWS: Optimal walking speed
RCT: Randomized controlled trial
RR: Risk ratio
SPPB: Short Physical Performance Battery
SSWS: Self-selected walking speed
STEMI: ST-segment elevation myocardial infarction
TIMI: Thrombolysis in Myocardial Infarction
TRIUMPH: Translational Research Investigating Underlying Disparities in Recovery from Acute Myocardial Infarction: Patients’ Health Status

## Abbreviations

ACS: Acute coronary syndrome
AMI: Acute myocardial infarction
ASCVD: Atherosclerotic cardiovascular disease
BNP: B-type natriuretic peptide
CHF: Chronic heart failure
CR: Cardiac rehabilitation
CV: Cardiovascular
ESC: European Society of Cardiology
GRACE: Global Registry of Acute Coronary Events
HF: Heart failure
HR: Hazard ratio
LRI: Locomotor Rehabilitation Index
LVEF: Left ventricular ejection fraction
MACE: Major adverse cardiovascular events
MACCE: Major adverse cardiac and cerebrovascular events
MI: Myocardial infarction
OR: Odds ratio
OWS: Optimal walking speed
RCT: Randomized controlled trial
RR: Risk ratio
SPPB: Short Physical Performance Battery
SSWS: Self-selected walking speed
STEMI: ST-segment elevation myocardial infarction
TIMI: Thrombolysis in Myocardial Infarction
TRIUMPH: Translational Research Investigating Underlying Disparities in Recovery from Acute Myocardial Infarction: Patients’ Health Status
